# NF‐κB/NLRP3 inflammasome axis and risk of Parkinson's disease in Type 2 diabetes mellitus: A narrative review and new perspective

**DOI:** 10.1111/jcmm.17784

**Published:** 2023-05-21

**Authors:** Mohammed Alrouji, Hayder M. Al‐kuraishy, Ali I. Al‐Gareeb, Athanasios Alexiou, Marios Papadakis, Majid S. Jabir, Hebatallah M. Saad, Gaber El‐Saber Batiha

**Affiliations:** ^1^ Department of Clinical Laboratory Sciences, College of Applied Medical Sciences Shaqra University Shaqra Saudi Arabia; ^2^ Department of Clinical Pharmacology and Medicine, College of Medicine ALmustansiriyia University Baghdad Iraq; ^3^ Department of Science and Engineering Novel Global Community Educational Foundation Hebersham New South Wales Australia; ^4^ AFNP Med Wien Austria; ^5^ Department of Surgery II University Hospital Witten‐Herdecke, University of Witten‐Herdecke Wuppertal Germany; ^6^ Applied Science Department University of Technology Baghdad Iraq; ^7^ Department of Pathology, Faculty of Veterinary Medicine Matrouh University Matrouh Egypt; ^8^ Department of Pharmacology and Therapeutics, Faculty of Veterinary Medicine Damanhour University Damanhour Egypt

**Keywords:** NF‐κB, NLRP3 inflammasome, Parkinson disease, T2DM

## Abstract

Parkinson's disease (PD) is the second most common neurodegenerative disease after Alzheimer's disease (AD). Genetic predisposition and immune dysfunction are involved in the pathogenesis of PD. Notably, peripheral inflammatory disorders and neuroinflammation are associated with PD neuropathology. Type 2 diabetes mellitus (T2DM) is associated with inflammatory disorders due to hyperglycaemia‐induced oxidative stress and the release of pro‐inflammatory cytokines. Particularly, insulin resistance (IR) in T2DM promotes the degeneration of dopaminergic neurons in the substantia nigra (SN). Thus, T2DM‐induced inflammatory disorders predispose to the development and progression of PD, and their targeting may reduce PD risk in T2DM. Therefore, this narrative review aims to find the potential link between T2DM and PD by investigating the role of inflammatory signalling pathways, mainly the nuclear factor kappa B (NF‐κB) and the nod‐like receptor pyrin 3 (NLRP3) inflammasome. NF‐κB is implicated in the pathogenesis of T2DM, and activation of NF‐κB with induction of neuronal apoptosis was also confirmed in PD patients. Systemic activation of NLRP3 inflammasome promotes the accumulation of α‐synuclein and degeneration of dopaminergic neurons in the SN. Increasing α‐synuclein in PD patients enhances NLRP3 inflammasome activation and the release of interleukin (IL)‐1β followed by the development of systemic inflammation and neuroinflammation. In conclusion, activation of the NF‐κB/NLRP3 inflammasome axis in T2DM patients could be the causal pathway in the development of PD. The inflammatory mechanisms triggered by activated NLRP3 inflammasome lead to pancreatic β‐cell dysfunction and the development of T2DM. Therefore, attenuation of inflammatory changes by inhibiting the NF‐κB/NLRP3 inflammasome axis in the early T2DM may reduce future PD risk.

## 1 INTRODUCTION

Parkinson's disease (PD) is the second most common neurodegenerative disease after Alzheimer's disease (AD).^1^ PD was initially identified in 1817 by Doctor James Parkinson who described shaking palsy.^2^ PD is progressing due to the loss of dopaminergic neurons in the substantia nigra (SN) followed by a considerable dopamine deficiency in the caudate nucleus and putamen.^1^ These changes lead to the development of motor dysfunctions including rigidity, resting tremors, bradykinesia and walking difficulty.^3^ In addition, numerous non‐motor disorders are developed including apathy, depression, anxiety, autonomic disorders, dementia, neuropsychiatric disorders, cognitive dysfunction and sleep disturbances.^4^ The incidence of PD in the general population is 0.3% and reaches 4% above the age of 80 years.^5^ The mean age of PD onset is around 60 years; though, new‐onset PD may develop in the younger age group of 20–50 years.^6^ The annual incidence of PD is 8–18 per 100,000.^6^ PD is more common in China and it is expected that 50% of total PD patients will be in this country by 2030.^7^ Higher incidence of PD could be related to population growth and aging, genetic predisposition, lifestyle changes, single gene polymorphism and environmental pollution. Moreover, the improved diagnostic methods also led to better identification of disease, especially in early stages.^7^, ^8^

**FIGURE 1:**
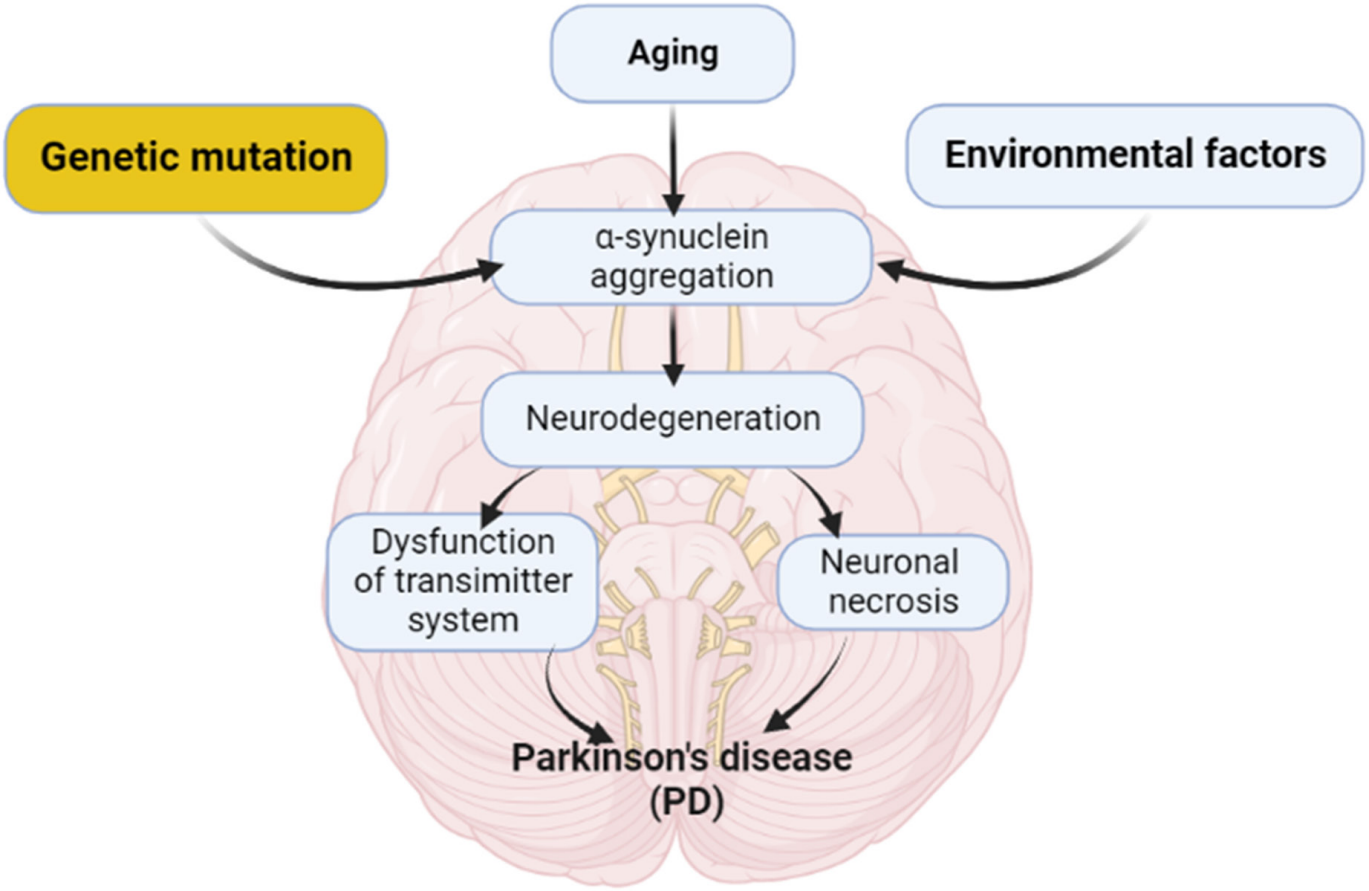
Pathophysiology of PD.

Males are more affected by PD than females with a ratio of 3:2.^9^ In 2040, the number of PD patients will extend to 14 million people with a risk for the progress of the Parkinson's pandemic.^9^ PD may be genetic or non‐genetic due to exposure to pesticides, and manganese.^10^, ^11^ The neuropathological characteristic of PD is the deposition of Lewy bodies from aggregated α‐synuclein (Figure 1).^12^

Deposition of α‐synuclein is not limited to the SN but throughout the entire brain such as the autonomic nervous system (ANS).^12^ Deposition of α‐synuclein is progressive for many years before the development of a symptomatic period.^12^ Deposition of α‐synuclein is starting initially in the ANS mainly in the dorsal motor nucleus of glossopharyngeal and vagus nerves and then spreads to the other brain areas in stage I PD.^13^ Stage of α‐synucleinopathy is characterized by spreading to the brain stem area including medulla oblongata, locus coeruleus and pontine tegmentum. In stage α‐synucleinopathy, the SN is affected. During stage IV α‐synucleinopathy, there is profound degeneration of dopaminergic neurons in the SN and the pathology of Lewy bodies extends to the temporal cortex. In the advanced V and VI stages, Lewy bodies are highly deposited in the neocortex leading to the development of cognitive dysfunction.^13^ These findings suggest that PD neuropathology is not limited to SN degeneration. Notably, in the prodromal phase, non‐motor symptoms including anosmia, constipation, sleep disorders and depression are developing before dopaminergic degeneration in the SN. Following the development of motor symptoms due to dopaminergic degeneration in the SN, cognitive dysfunctions are propagated due to the involvement of the temporal cortex.^13^, ^14^, ^15^ Besides, PD is associated with the progression of various inflammatory disorders which are linked with the progression of PD neuropathology.^16^

Genetic and environmental factors have been described as of major importance in T2DM development such as obesity, which is directly correlated with the development of insulin resistance (IR) and inflammatory state in metabolic activated adipose tissue.^17^ Inflammatory responses may have a dual role in T2DM, as it may have either a causal relationship leading to IR or may be intensified by the hyperglycaemic state, resulting in T2DM complications.^18^ Hyperglycaemia is the major risk factor for microvascular complications and reduction in glycated haemoglobin (HbA1c) decreases the incidence of retinopathy, nephropathy and neuropathy.^19^ For every 1% decrement in HbA1c, the incidence of microvascular complications is reduced by about 25%–35%.^20^ The incidence of T2DM is increasing worldwide and has become a significant public health problem. It is associated with mortality and significant morbidity, including neurological disability.^21^ Although the effects of diabetes on the peripheral nervous system are well established, its effects on higher mental and specific neurological functions are often overlooked. Various studies reported that T2DM could be a risk factor for the development of PD.^22^, ^23^ However, the underlying mechanisms linking T2DM and PD are not fully elucidated. Therefore, this narrative review aimed to find the potential link between T2DM and PD regarding the potential role of inflammatory signalling pathways.

## 2 T2DM AND INFLAMMATORY DISORDERS

T2DM is associated with inflammatory disorders and end‐organ damage due to hyperglycaemia‐induced oxidative stress and the release of pro‐inflammatory cytokines.^24^ IR and relative insulin deficiency due to pancreatic β‐cell dysfunction is the major feature of T2DM.^25^ The triggering of inflammatory disorders in T2DM is not fully elucidated, although it has been shown that immune cell dysregulation and infiltration into adipose tissue promote the expression of pro‐inflammatory cytokines with the development of systemic inflammation.^26^ Prolonged low‐grade inflammation in T2DM due to hyperglycaemia and adipose tissue activation leads to the development of IR and the propagation of several related complications.^27^ Inflammatory disorders seem to play a critical role in the progression of IR, T2DM and systemic complications.^26^, ^27^ Both hypoglycaemia and hyperglycaemia as well as glucose variability (daily changes of blood glucose with controlled glycated haemoglobin) trigger oxidative stress which in turn promotes inflammatory disorders.^28^

**FIGURE 2:**
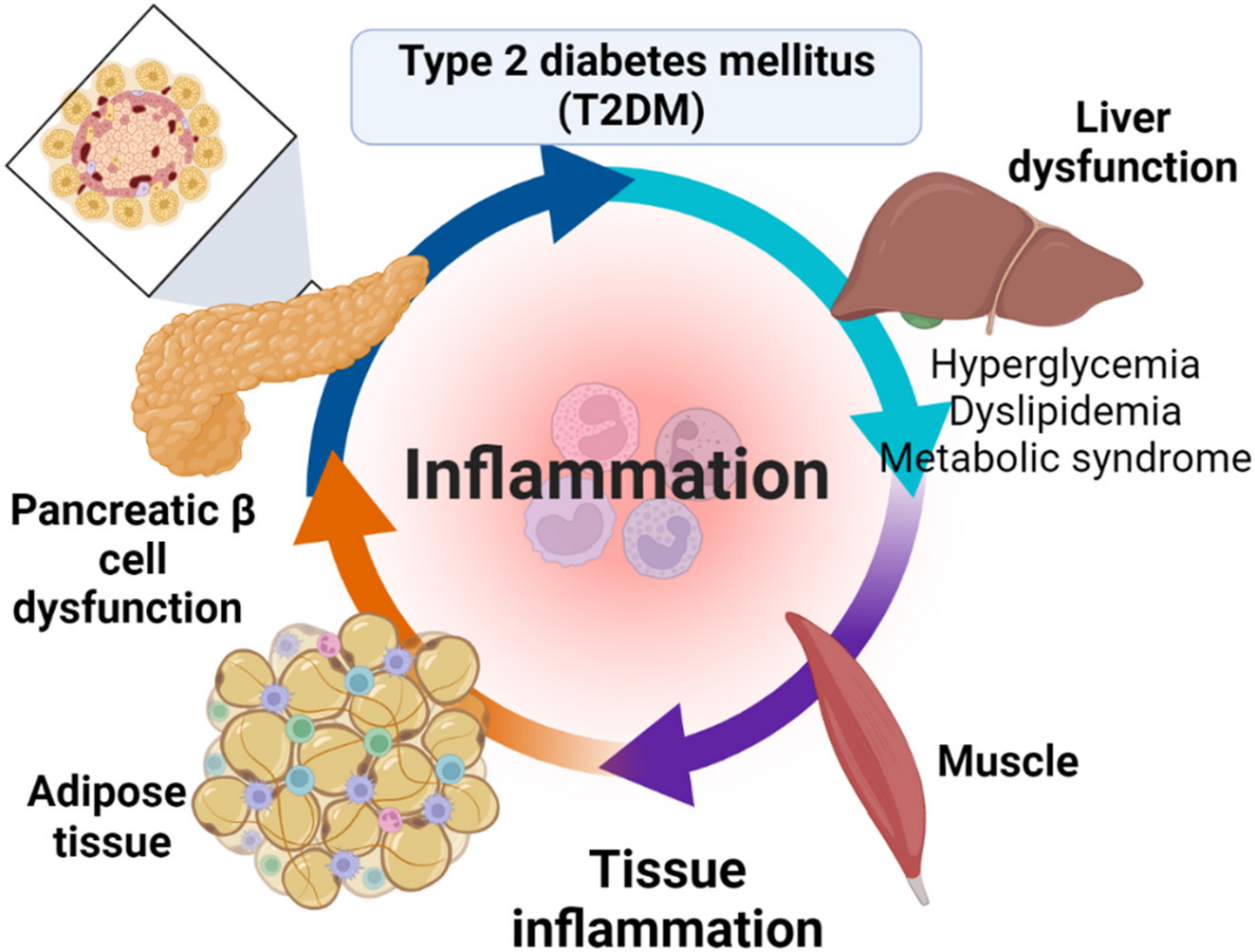
T2DM and associated inflammatory disorders.

Besides, environmental and genetic factors such as stress, diet and smoking are engaged in the activation of chronic inflammation in T2DM.^28^ A population‐based study illustrated that the risk of T2DM is increased with the presence of systemic inflammatory disorders.^26^ A systematic review and meta‐analysis on 10 prospective studies and 22 cohort studies showed that higher serum levels of IL‐6 and C reactive protein (CRP) increase the risk for the development of T2DM.^29^ Thus, anti‐inflammatory agents may reduce the risk.^25^ These observations suggest a close relationship between T2DM and immunoinflammatory disorders in a feedback loop (Figure 2).

### 2.1 PD and inflammatory disorders

Progression of PD is associated with high inflammatory changes and systemic inflammatory disorders.^30^ For example, PD is more common in inflammatory bowel diseases (IBDs), as shown by a systematic review illustrating that PD is more common in patients with Crohn's disease and ulcerative colitis.^30^ A retrospective study indicated that patients with IBDs had a higher risk for the development of PD.^30^ Numerous cytokines like interleukin‐1β (IL‐1β) and tumour necrosis factor‐alpha (TNF‐α) which are higher in IBDs and other inflammatory disorders are involved in the pathogenesis of PD.^30^ These data indicated that higher inflammatory changes may increase for development of PD.

Of note, dysfunction of the immune system with genetic susceptibility impairs cellular immune responses in PD.^31^ Deregulation of the innate/adaptive immune response is implicated in the development of neurodegenerative diseases including PD.^31^ In PD, both central and peripheral immune responses are disturbed with increased risk for the development of an autoimmune response.^31^ A 33% overall excess risk of PD was noted among patients with autoimmune disorders like Graves's disease, multiple sclerosis, pernicious anaemia and polymyalgia rheumatica; the risk being increased during the first 10 years of follow‐up after hospitalisation of autoimmune disorders.^32^ Of note, leucine‐rich repeat kinase 2 (LRRK2) which regulates B‐lymphocyte function, is regarded as a potential link between cell‐mediated immunity and PD neuropathology.^33^ A number of disease genes have been recognized as modulators of immune functions in PD and confirmation is rising for the role of viral infections, pesticides exposure and changes in gut microbiota in disease pathogenesis through modulation of the immune response.^8^ Therefore, genetic predisposition and immune dysfunction are intricate in the pathogenesis of PD.

**FIGURE 3:**
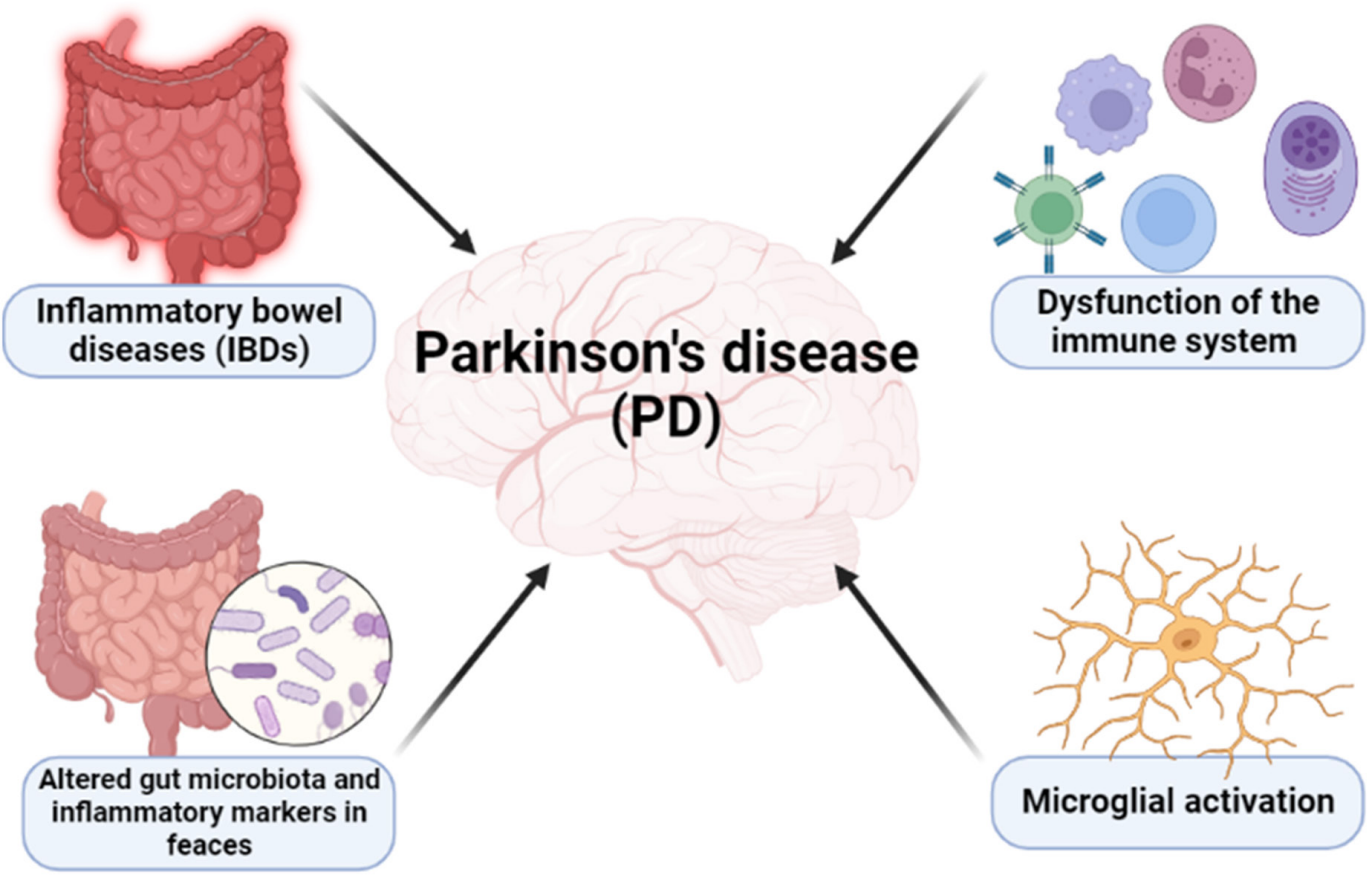
PD and inflammatory disorders.

Peripheral inflammatory biomarkers may be augmented and correlated with motor severity in PD patients.^34^ A case–control study on 58 PD patients compared to 20 healthy controls showed that IL‐1β, TNF‐α, IL‐6, CRP and IL‐12 are increased in PD patients compared to healthy controls.^34^ There was no positive correlation between the levels of inflammatory biomarkers and non‐motor symptoms in PD patients.^34^ Meanwhile, Chen et al.^35^ revealed that there was an aberrant alteration in inflammatory cytokines in the cerebrospinal fluid (CSF) in patients with neurodegenerative disorders including PD. Moreover, an abnormal immune response and microglia hyper‐activation are linked with the degeneration of dopaminergic neurons in the SN.^36^ These findings indicated that the progression of PD is highly correlated with the severity of peripheral and local inflammatory disorders (Figure 3).

### 2.2 T2DM and the risk of PD

Interestingly, PD is linked with T2DM, particularly in aetiology, epidemiology and pathogenesis. T2DM is regarded as a risk factor for PD. Notably, subjects with impaired glucose tolerance were shown to be at higher risk for the development of cognitive decline.^23^ Thus, T2DM patients may have PD‐like symptoms such as motor dysfunction as both PD and T2DM share overlapping pathology. Therefore, T2DM may predispose toward a PD‐like pathology, and, when present in PD patients, can induce a more aggressive phenotype.^22^

Various studies revealed the association between T2DM and PD (Table 1). Evidences from epidemiological and observational studies showed a potential controversy regarding the association between T2DM and PD.^23^, ^37^, ^38^, ^39^, ^40^, ^41^ In an observational study, T2DM patients appear to be at increased risk of developing PD, as well as experiencing faster progression and a more severe phenotype of PD, with the effects being potentially mediated by several common cellular pathways. The insulin signalling pathway, for example, may be responsible for neurodegeneration via insulin dysregulation, aggregation of amyloids, neuroinflammation, mitochondrial dysfunction and altered synaptic plasticity.^37^ However, there are studies that showed the opposite or no relation between these diseases.^40^, ^41^

**TABLE 1:** The association between T2DM and PD.

Study type	Findings	Ref.
A cohort study	Increased rates of subsequent PD following T2DM	De Pablo‐Fernandez et al.^23^
A review	T2DM augments PD risk	Cheong et al.^37^
A systematic review	T2DM increases PD risk	Camargo Maluf et al.^38^
A prospective study	T2DM is associated with an increased risk of PD	Hu et al.^39^
Meta‐analysis of case–control studies	T2DM patients have a low risk for the development of PD	Lu et al.^40^
A case–control study	No association between T2DM and PD	Savica et al.^41^
A case–control study	T2DM may predispose toward a PD‐like pathology, and when present in patients with PD, can induce a more aggressive phenotype	Pagano et al.^22^
A population‐based study	T2DM increased the risk of PD during a mean follow‐up of 7.3 years	Yang et al.^48^
A population‐based cohort study	T2DM patients increase PD risk mainly in younger women	Sun et al.^51^
A population‐based cohort study	Prediabetes and T2DM increase PD risk mainly in younger women	Sánchez‐Gómez et al.^58^

Of note, IR in T2DM promotes the degeneration of dopaminergic neurons in the SN and other PD neuropathological processes since; insulin signalling regulates dopaminergic synaptic plasticity and neurotransmission in the SN.^23^ An experimental study observed that T2DM mice not only showed IR and impairment of insulin signalling in the pancreatic β cells and liver but also in the midbrain DNs.^42^ These changes are developed due to the deposition of α‐synuclein and associated endoplasmic reticulum stress.^42^ Therefore, metabolic inflammation exacerbates the degeneration of DNs and contributes to the degeneration of DNs and the progress of PD.

Indeed, preclinical and clinical evidence indicated that T2DM is associated with an increased risk for the development of PD.^22^, ^43^ The connection between T2DM and risk for the development of PD was primarily reported in 1993 by Sanyk, who showed that PD patients with T2DM had severe motor dysfunction and respond less to PD pharmacotherapies.^44^ Afterward, Marques et al.^44^ illustrated that PD patients had abnormal glucose tolerance which could be due to the development of dysautonomia and impairment of insulin response. It has been revealed that degeneration of dopaminergic neurons in the SN influences glycaemic control since SN regulates the feeding behaviour during hypoglycaemia.^45^ This conclusion anticipated the mutual relationship between T2DM and PD.^37^, ^46^

A prospective study on 25 PD patients with T2DM compared to 25 PD patients without T2DM followed for 36 months showed that T2DM patients with PD experience more motor dysfunctions.^22^ The incidence of PD in the general population is 0.03% though this percentage is augmented to 18% in T2DM patients.^47^ Thus, T2DM predisposes to more complications and a more aggressive phenotype of PD. A population‐based study performed by Yang et al.^48^ showed that T2DM augments the risk for PD. A meta‐analysis demonstrated that T2DM increases the risk for PD by 38%.^49^ As well, PD and T2DM share matching dysregulated pathways like genetic vulnerability and exposure to environmental risk factors.^43^ It has been observed that systemic exposure to environmental toxins induces several pathological features of PD such as mitochondrial dysfunction, oxidative stress, inflammation, and α‐synuclein misfolding, thereby indicating environmental exposure as a contributor to the PD disease process. Exposure to environmental toxins may interact with polymorphisms in genes involved in free radical scavenging and protein degradation, leading to an increased risk to develop PD in some individuals.^50^ The progress of peripheral IR and brain IR due to mitochondrial dysfunction, endoplasmic reticulum stress and chronic inflammation alterations could be a possible pathway linking PD and T2DM.^43^

The probable link between PD and T2DM is the age factor, as both of these diseases are amplified by aging due to mitochondrial dysfunction and endoplasmic reticulum stress.^51^ A retrospective study showed that T2DM patients increase PD risk mainly in younger women.^51^ In addition, dysregulation of the brain insulin signalling pathway in T2DM reduces dopaminergic activity in the SN.^52^ Brain IR is augmented in both PD and T2DM patients compared to the control.^53^ These findings emphasized that T2DM increases the risk for the progression of PD. Notably, insulin signalling has been found to be de‐sensitized in the brains of PD patients, and drugs that can re‐sensitize insulin signalling may control disease progression. Insulin signalling plays important roles in neuronal growth, synaptic development, energy utilisation, mitogenesis, inhibition of apoptosis and more. Insulin binds to the α‐subunit of the receptor. This activates the tyrosine kinase phosphorylation of the β‐subunit.^54^ Preclinical studies have shown that animal models of AD and PD show impaired insulin signalling and a range of downstream effects that contribute to the pathology.^54^ Thus, improvement of insulin signalling in the brains of AD or PD patients has clear disease‐modifying effects. Not only are symptoms such as attention, memory impairments, or other cognitive impairments much reduced in AD and PD patients, and motor coordination improved in PD patients, but the improvements long outlast drug treatment duration and support the rationale for repurposing anti‐diabetic drugs for PD treatment.^55^, ^56^ At the cellular level, long‐term elevated levels of glucose have been shown to lead to nigrostriatal degeneration and an increase of α‐synuclein accumulation through induction of neuroinflammation in PD models.^57^

Furthermore, pre‐diabetes also boosts the risk for the development of PD.^17^, ^58^ A retrospective, cohort‐population‐based study comprising 281,153 patients with T2DM and 266,379 with prediabetes and a reference cohort of 2,556,928 subjects investigated from 2006 to 2018 illustrated that prediabetic patients are associated with subsequent PD development.^58^ Nevertheless, the use of oral hypoglycaemic agents in T2DM patients with anti‐inflammatory properties like metformin may reduce PD risk in T2DM patients.^59^, ^60^

It has been shown that T2DM accelerates the development of cognitive dysfunction and motor deficits in PD via the reduction of the accessibility and expression of dopamine transporters.^22^ Further on, Chung et al.^61^ established that T2DM has detrimental effects on the dopaminergic transporters and brain cortical thickness. However, Bohnen et al.^62^ illustrated that T2DM is independently associated with the development of the severe form of PD via a mechanism other than PD‐specific neurodegeneration like dopamine depletion and NS degeneration. Consequently, PD appears to be more brutal when linked with comorbid T2DM as PD patients with T2DM have more frontotemporal cortical atrophy compared with PD patients without T2DM.^62^ Of note, tau protein which is a biomarker of NBDs as in AD and PD is augmented in the CSF of PD patients with T2DM compared to PD without T2DM.^22^, ^45^ Peripheral IR and hyperglycaemia provoke the development of chronic inflammation, microvascular dysfunction, neuroinflammation and impairment of the blood–brain barrier (BBB) function.^63^ Notably, IR increases glutamate excitotoxicity and the development of synaptic dysfunction in the SN with the development of PD.^64^ Brain IR promotes as well abnormal protein aggregation and impairment of amyloid protein clearance.^63^ It has been reported that insulin receptors that control the expression of dopamine transporters, normalize dopamine release and reduce dopamine turnover are highly expressed by SN.^65^ Thus, the expression of mRNA of neuronal insulin receptors in the SN is highly expressed in post‐mortem PD patients compared to the controls.^66^

An additional possible mechanism for the connection between PD and T2DM is the accumulation of amyloid proteins and misfolded proteins. Of interest, the accumulation of amyloid polypeptide in the pancreatic β cells and deposition of α‐synuclein with the formation of Lewy bodies are the hallmarks of T2DM and PD, respectively.^67^, ^68^ In addition, α‐synuclein aggregates were detected in the pancreatic β cells of PD patients adjacent to the accumulation site of amyloid peptides.^68^ The presence of α‐synuclein deposits in the pancreas of patients with synucleinopathies, as well as tau and amyloid beta (Aβ) deposits in the pancreatic tissue of patients with NDs highlighted the potential nexus. A study on the pancreas of 138 subjects with NDs or T2DM showed that prion protein, Aβ, α‐synuclein, amylin and tau proteins are highly expressed in the pancreas and locus coeruleus.^69^ Patients with a synucleinopathy showed the highest α‐synuclein pancreatic immunoreactivity compared to control subjects.^70^

These results proposed a shared mechanism between T2DM and PD outside the CNS. In T2DM patients, IR inhibits the insulin‐degrading enzyme (IDE) and increases the generation of α‐synuclein with an increased risk for the development of PD.^69^ In sequence, α‐synuclein in the pancreatic β cells inhibits insulin release and development of IR.^67^ Recently, pathological α‐synuclein deposits were found in the pancreatic β cells of 93% of subjects with PD, in 68% of subjects with T2DM following neuropathological examination and in 17% of control subjects.^70^ Additionally, increased accumulation, aggregation and phosphorylation of α‐synuclein was observed in the pancreatic islets of non‐human primate models of spontaneous T2DM.^71^ These findings support links between α‐synuclein and T2DM which is characterized by both a loss of insulin sensitivity in peripheral tissues such as the skeletal muscle and impaired insulin secretion by pancreatic β cells.

Additionally, prolonged effects of T2DM promote the formation of advance glycation end‐product (AGE) in various tissues including CNS, mainly in the SN.^72^ AGE interrelates with AGE receptors (AGER) near to the accumulation sites of Lewy bodies and α‐synuclein.^73^ AGE augments the deposition and formation of a more toxic α‐synuclein which resists the degradation by ubiquitin and proteases enzymes.^74^ Hence, the generation of AGE also increases the risk for the development of other neurodegenerative disorders like AD through the induction of oxidative stress and associated neuroinflammation.^75^ Furthermore, mitochondrial dysfunction in PD triggers the development of oxidative stress and progressive neuronal death.^75^ Mitochondrial dysfunction might be a probable way to connect T2DM and PD which are associated with pancreatic β‐cell dysfunction and loss of dopaminergic neurons in the SN.^73^, ^76^

Moreover, microglia cells play a critical role in synaptic regeneration by encouraging the release of neuroprotective factors.^69^ In turn, over‐activated microglia cells enhance the development of neuroinflammation in PD through the release of pro‐inflammatory cytokines.^77^ Early microglia activation is associated with neuroprotection while prolonged microglia activation induces neuroinflammation by triggering the stimulation of inflammatory signalling pathways.^78^ Prolonged microglia activation increases the risk for the progression of PD^77^ and T2DM.^79^ Particularly, obesity‐induced chronic inflammation promotes microglia activation that could contribute to the development of the metabolic syndrome and T2DM, potentially triggering neurological disorders.^72^

**FIGURE 4:**
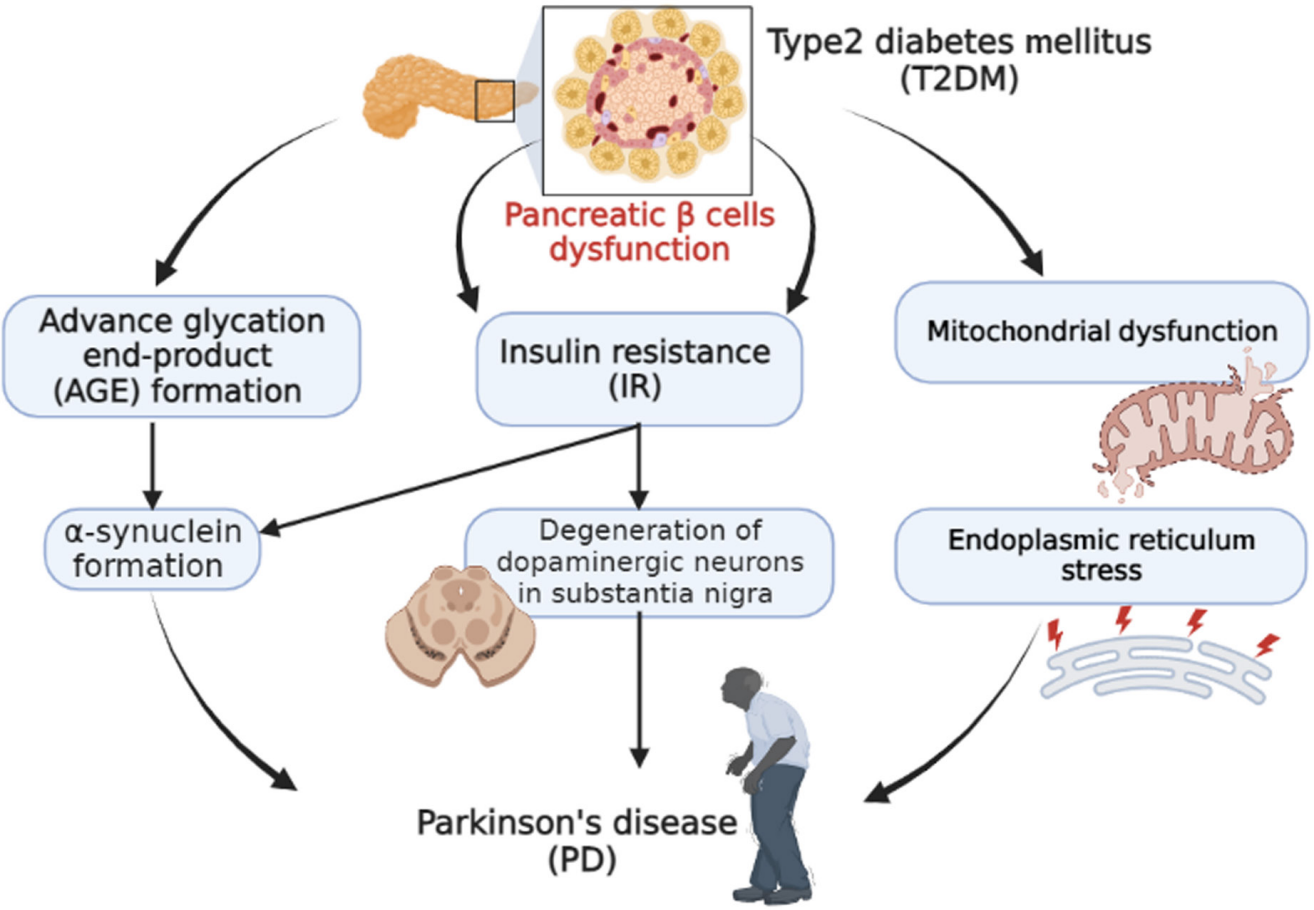
The mutual relationship between T2DM and PD.

These results have anticipated the mutual relationship between T2DM and PD; in particular, T2DM could be a risk factor in the development of PD (Figure 4).

## 3 NF‐κB/NLRP3 INFLAMMASOME AXIS

Inflammatory signalling pathways mediated by the nuclear factor kappa B (NF‐κB), nod‐like receptor pyrin 3 (NLRP3) inflammasome and mitogen‐activated protein kinase (MAPK), as well as other signalling pathways, are involved in the pathogenesis of T2DM and PD.^80^, ^81^

### 3.1 NF‐κB

NF‐κB is a DNA‐binding protein required for the transcription of many pro‐inflammatory cytokines and chemokines. NF‐κB plays a major role in coordinating the innate and adaptive immunity, cellular proliferation, apoptosis and development. NF‐κB is under the control of extracellular stimuli, it is inhibited by an inhibitor of κB (IκB) which sequesters NF‐κB in the cytosol and prevents its nuclear localisation.^82^ Cytokines inhibit IκB with subsequent nuclear translocation and activation of NF‐κB and propagation of consequent transcription of many inflammatory genes.^83^

NF‐κB is involved in the early pathological events of T2DM.^84^ Inhibition of NF‐κB by salicylate improves glucose control and insulin sensitivity in T2DM patients.^85^ Therefore, higher activation of NF‐κB is linked with more diabetic complications and IR.^85^ In addition, T2DM‐induced oxidative stress promotes the activation of NF‐κB with increased inflammatory processes.^86^ NF‐κB is also activated by reactive oxygen species (ROS) and pro‐inflammatory cytokines, thus, chronic low‐grade inflammatory disorders and oxidative disorders in T2DM could be the possible mechanism for the activation of NF‐κB.^87^ It has been reported that the over‐activation of NF‐κB is linked with the development of metabolic disorders in T2DM patients including obesity and atherosclerosis.^86^ Besides, over‐expression of the NF‐κB activator IKKβ in the liver results in the development of defective insulin signalling with the development of peripheral IR.^88^ As well, IKKβ activators promote the expression of IL‐6 with subsequent propagation of inflammatory disorders, supporting the role of NF‐κB in the development of IR.^89^ Deletion of IKKβ in mice protects from the development of peripheral IR. However, increased expression of IKKβ in the liver is linked with high‐fat diet‐induced IR.^90^ As well, hyperglycaemia activates IKKβ leading to impairment of insulin signalling and development of endothelial dysfunction (ED) by inhibiting the nitric oxide signalling pathway.^89^ These findings involve NF‐κB in the pathogenesis of T2DM which is characterized by higher levels of activated NF‐κB.^91^

On the other hand, NF‐κB is also involved in the pathogenesis of PD through the induction of inflammation‐mediated degeneration of dopaminergic neurons in the SN.^92^ Immune dysregulation due to aging promotes the activation of NF‐κB with subsequent neuronal injury and neuroinflammation with the development of PD.^92^ Findings from post‐mortem studies suggest the role of NF‐κB in the degeneration of dopaminergic neurons in the SN. NF‐κB inhibition suppresses pro‐inflammatory cytokine expression, protecting dopaminergic neurons and improving motor activity.^93^ Ghosh et al.^93^ illustrated that selective inhibition of NF‐κB prevents the degeneration of dopaminergic neurons in the SN in the mouse model of PD. Likewise, targeting the NF‐κB pathway in murine models of PD may prevent PD progression.^94^ Notably, different drugs and herbals like pioglitazone, salmeterol and curcumin can hinder the degeneration of dopaminergic neurons in the SN by inhibiting NF‐κB which is involved in the progression of neuroinflammation and injury of dopaminergic neurons.^94^ A recent finding evidenced that α‐synuclein released from injured dopaminergic neurons triggers the activation of NF‐κB and the release of pro‐inflammatory cytokines with further aggravation of dopaminergic neurons in a positive‐loop.^95^ These findings proposed that NF‐κB could be a therapeutic target in the management of PD. Remarkably, the Aβ_1–42_ level in the CSF is reduced and not correlated with motor dysfunction in PD patients compared to the controls.^96^ However, Shi et al.^97^ revealed that the Aβ_1–42_ level in the CSF is increased and correlated with the severity of PD. Nevertheless, Aβ_1–42_ inhibits BBB P‐glycoprotein through induction of NF‐κB with further reduction in the clearance of Aβ_1–42_.^98^ Thus, NF‐κB not only induces degeneration of dopaminergic neurons in the SN but also increases PD severity through the accumulation of Aβ_1–42_ and α‐synuclein.

Furthermore, dipeptidyl peptidase 4 (DPP4) inhibitors like vildagliptin reduce the expression of NF‐κB and the associated neuroinflammation, apoptosis, and oxidative stress in dopaminergic neurons of SN. DPP4 inhibitors also inhibit the expression of the RAGE, which act as a receptor for inflammatory S100/calgranulins and high mobility group box 1 (HMGB1), linking RAGE to both the consequences and causes of T2DM.^99^ This finding suggests that vildagliptin cuts the cross‐talk between PD and T2DM by inhibiting the NF‐κB signalling pathway.

**FIGURE 5:**
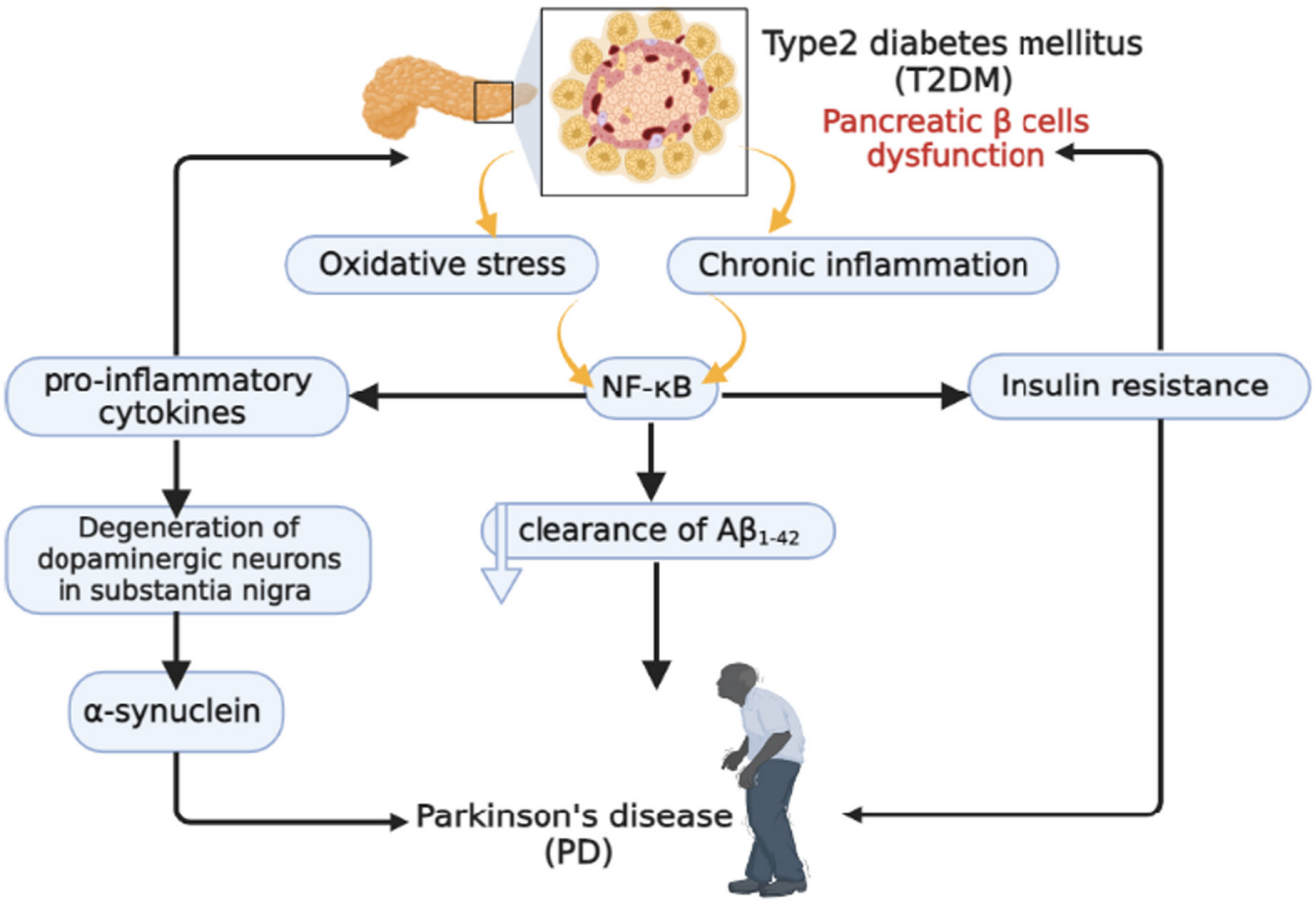
NF‐κB signalling pathway is activated in both T2DM and PD.

Taken together, the NF‐κB signalling pathway which is activated in both PD and T2DM might be a possible link in the development of PD in T2DM patients (Figure 5).

### 3.2 NLRP3 inflammasome

NLRP3 inflammasome is the nucleotide‐binding domain and the leucine‐rich repeat‐containing family and pyrin family which form a multiprotein complex. The main function of NLRP3 inflammasome is the activation of caspase‐1 and the consequent maturation of IL‐1β and IL‐18.^100^ The NLRP3 inflammasome is activated by different stimuli including alternative and non‐canonical pathways.^100^ The NLRP3 inflammasome is activated by NF‐κB and sphingosine‐1 phosphate.^101^, ^102^ The NLRP3 inflammasome is a metabolic sensor for thioredoxin‐reacting protein (TXNIP) and ROS which are implicated in the pathogenesis of T2DM.^103^ In this state, the activated NLRP3 inflammasome triggers the release of pro‐inflammatory cytokines which leads to pancreatic β‐cell dysfunction and the development of T2DM.^103^ IL‐1β, ROS and TXNIP are all implicated in the pathogenesis of T2DM. The NLRP3 inflammasome also drives IL‐1β maturation and secretion in another disease of metabolic dysregulation, gout. Thus, the NLRP3 inflammasome contributes to the pathogenesis of T2DM by functioning as a sensor for metabolic stress.^103^

Furthermore, the activated NLRP3 inflammasome promotes the development of IR and reduces glucose tolerance through modulation of gut microbiota and by increasing the expression of IL‐1β and IL‐18.^104^ IL‐1β and IL‐18 enhance glucose uptake by macrophages with the release of pro‐inflammatory cytokines and subsequent development of IR. In addition, these pro‐inflammatory cytokines promote the infiltration of macrophages into pancreatic β cells with succeeding injury and progression of pancreatic β‐cell dysfunction.^104^ Moreover, NLRP3 inflammasome‐induced pancreatic β‐cell pyroptosis could be a possible mechanism for the development of T2DM.^105^ Pyroptosis describes a pro‐inflammatory programmed cell death^106^ or is a caspase‐dependent and highly immunogenic form of cell death, characterized by the release of pro‐inflammatory cytokines and cellular contents.^54^ Consequently, pharmacological therapies targeted at pyroptosis‐related proteins including NLRP3 inflammasome components may serve as a promising strategy for acute pancreatitis treatment and prevention of T2DM.^106^

Furthermore, an experimental study confirmed that the activation of the NLRP3 inflammasome leads to the propagation of vascular inflammation and pro‐inflammatory phenotype in diabetic mice.^107^ In addition, the NLRP3 inflammasome is also involved in the development of ED which is the hallmark of diabetic complications.^107^ Of note, the development of ED in T2DM represents the first initial activator of the NLRP3 inflammasome leading to the release of pro‐inflammatory cytokines.^107^ Thus, T2DM is regarded as an inflammatory disease that provokes the development of numerous complications like nephropathy and retinopathy through NLRP3 inflammasome‐dependent mechanisms. Inhibition of the NLRP3 inflammasome by anti‐diabetic drugs like sulfonylurea, metformin, DPP4 inhibitors and thiazolidinedione can prevent the development of diabetic‐mediated complications.^108^ A case control study illustrated that the NLRP3 inflammasome and IL‐1β were higher in T2DM patients as compared to the controls.^109^ Of note, IL‐1β level in T2DM was correlated with the development of ED and atherosclerosis through the induction of adhesion molecules.^109^ In vitro study demonstrated that hyperglycaemia triggers the activation and release of pro‐inflammatory cytokines.^109^

**FIGURE 6:**
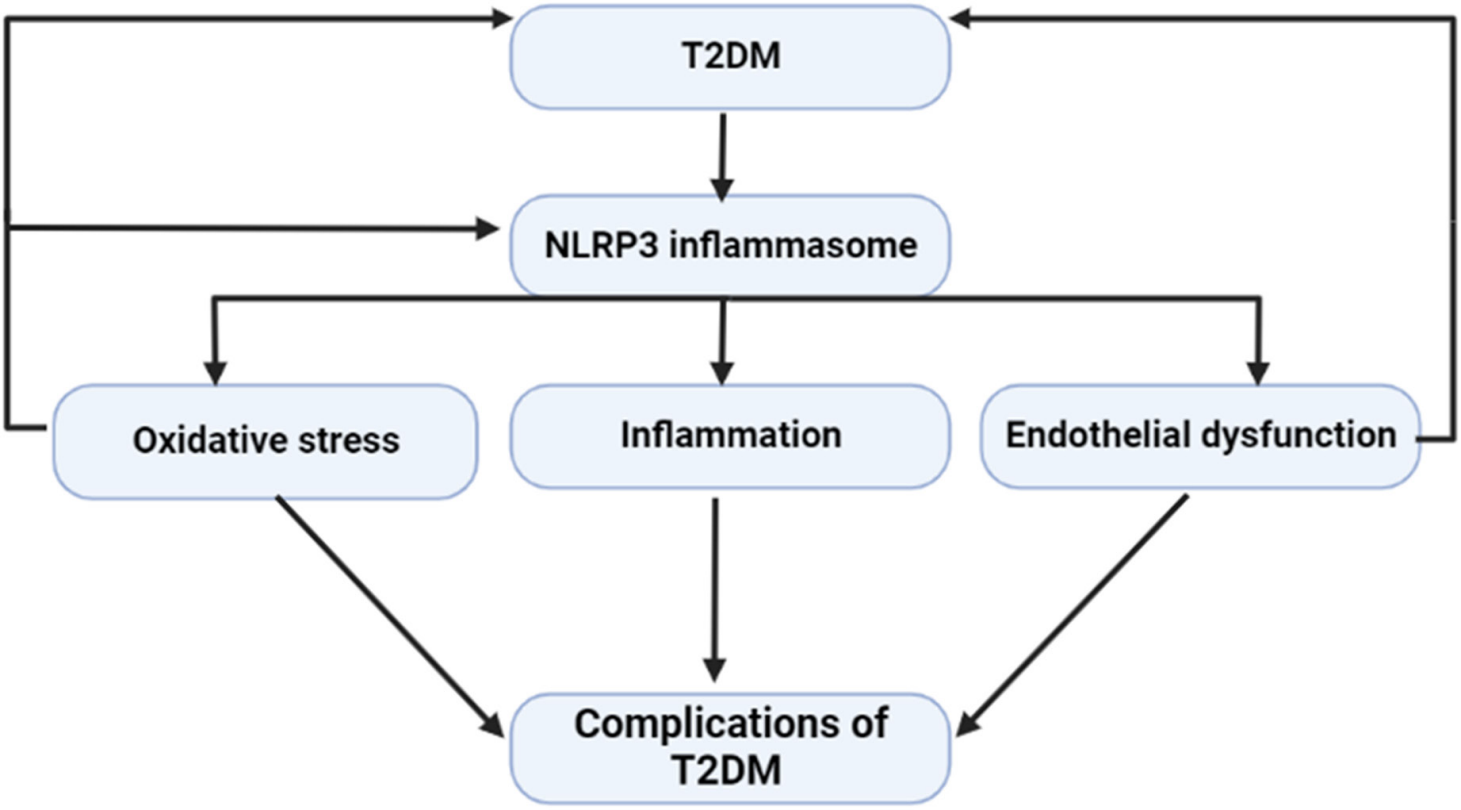
NLRP3 inflammasome is involved in the pathogenesis and complications of T2DM.

These findings indicated that the NLRP3 inflammasome is involved in the pathogenesis and complications of T2DM and targeting of NF‐κB and NLRP3 inflammasome signalling pathways by specific inhibitors could be effective in the management of T2DM (Figure 6).

On the other hand, the NLRP3 inflammasome is involved in the pathogenesis of PD.^110^ The NLRP3 inflammasome induces the release of pro‐inflammatory cytokines and the development of neuroinflammation and degeneration of dopaminergic neurons by induction of pyroptosis.^110^, ^111^ Moreover, accumulation of the α‐synuclein triggers the activation of the microglia with subsequent expression of the NLRP3 inflammasome in the SN.^110^ In addition, systemic activation of the NLRP3 inflammasome promotes the accumulation of α‐synuclein and the degeneration of dopaminergic neurons in the SN.^112^ A case–control study that included 67 PD patients compared to 24 healthy controls showed that the plasma levels of α‐synuclein, NLRP3 inflammasome, caspase‐1 and IL‐1β were increased in PD patients compared to healthy controls.^112^ Therefore, α‐synuclein, NLRP3 inflammasome and IL‐1β plasma levels could serve as biomarkers to monitor PD severity and progression. Different studies showed that higher levels of pro‐inflammatory cytokines in the CSF and plasma support the interaction between the brain and the immune system with the development of neuroinflammation and degeneration of dopaminergic neurons in PD.^113^, ^114^ It has been shown that IL‐1β plasma level was augmented in PD patients.^115^ These observations proposed that systemic inflammation via induction of neuroinflammation may lead to the degeneration of dopaminergic neurons and the development of PD.

**FIGURE 7:**
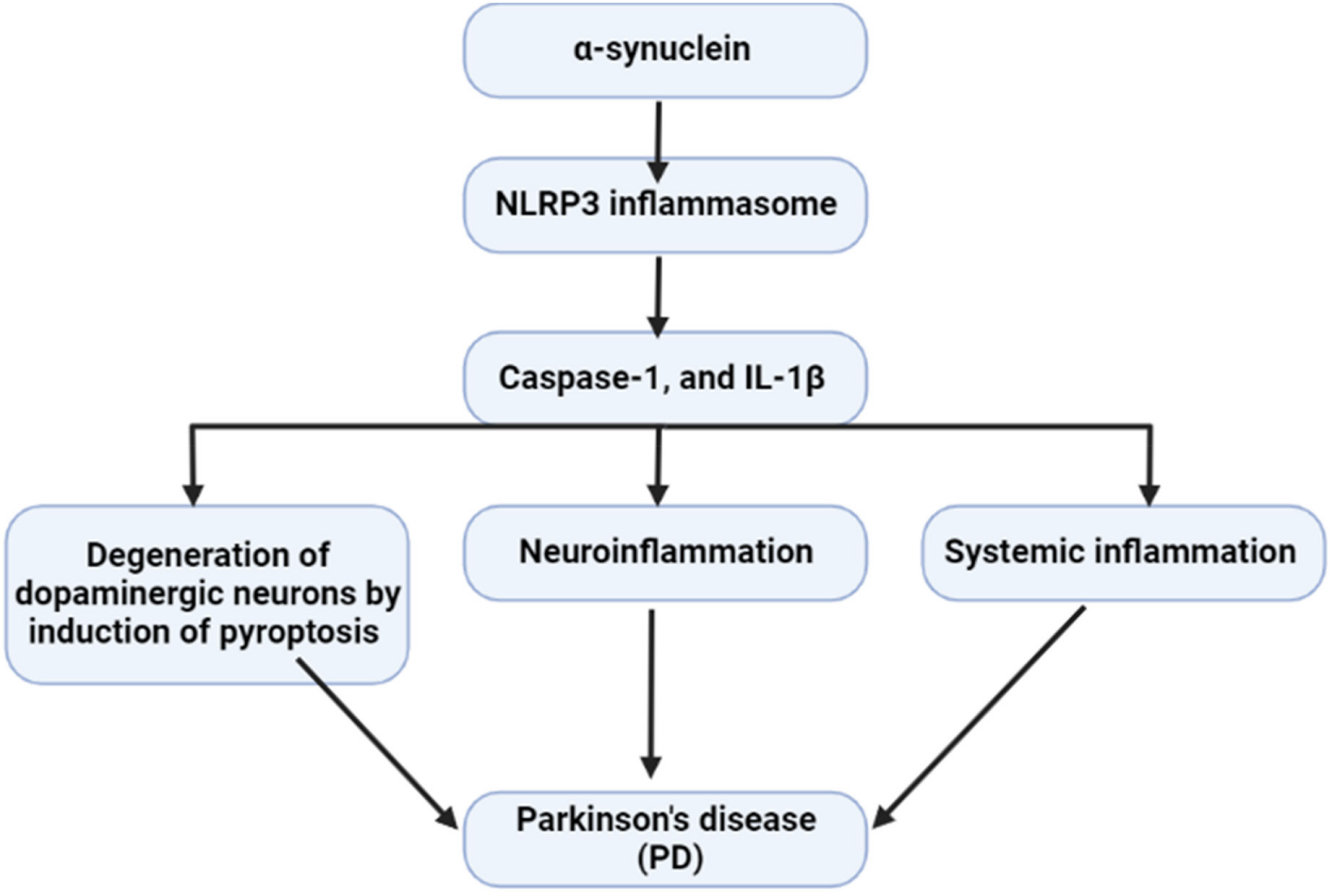
α‐synuclein triggers the activation of the NLRP3 inflammasome with subsequent release of IL‐1β and the development of systemic inflammation and neuroinflammation.

Furthermore, α‐synuclein plasma level which is a major constituent of Lewy bodies had been reported to be increased in PD patients compared to the healthy controls.^116^ In turn, α‐synuclein can trigger the activation of the NLRP3 inflammasome with subsequent release of IL‐1β and the development of systemic inflammation and neuroinflammation^117^ (Figure 7).

Accordingly, the NLRP3 inflammasome could be a potential link between PD and T2DM. A previous study revealed that T2DM was associated with higher α‐synuclein plasma levels which induce systemic inflammation and can cross BBB with induction of neuroinflammation through activation of NLRP3 inflammasome.^118^ A recent experimental study illustrated that chronic hyperglycaemia in T2DM may increase the risk of neuroinflammation and degeneration of dopaminergic neurons in PD through the activation of α‐synuclein.^57^ In addition, chronic hyperglycaemia in T2DM was shown to increase the deposition of α‐synuclein in the pancreatic β cells with aggravation of pancreatic β‐cells dysfunction and hyperglycaemia.^57^ Chronic hyperglycaemia can trigger oxidative stress which induces the release of pro‐inflammatory cytokines.^119^ These mediators can cross BBB causing the activation of the NLRP3 inflammasome and the deposition of α‐synuclein with the development of PD.^23^ Activation of the NLRP3 inflammasome in T2DM is associated with the development of brain IR which is involved in the degeneration of dopaminergic neurons in PD.^120^

**FIGURE 8:**
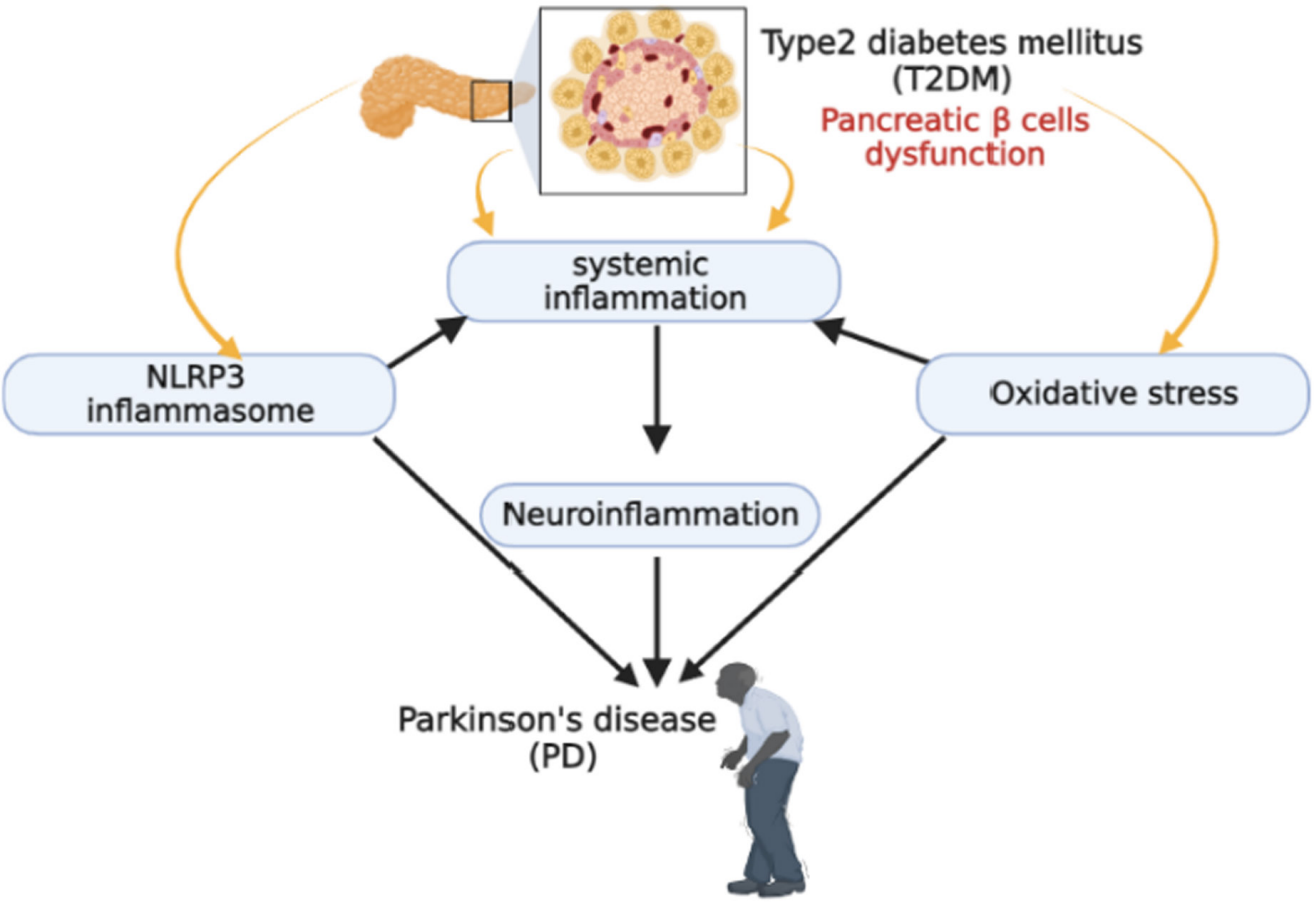
T2DM‐induced hyperglycaemia promotes the activation of the NLRP3 inflammasome and neuroinflammation leading to the development of PD.

These findings indicate that T2DM‐induced hyperglycaemia promotes the activation of the NLRP3 inflammasome with the propagation of systemic inflammation and the development of neuroinflammation and subsequent degeneration of dopaminergic neurons in the SN (Figure 8).

Taken together, activation of the NF‐κB/NLRP3 inflammasome axis in T2DM patients could be the causal pathway for the development of PD. Targeting of this axis by anti‐inflammatory agents may prevent or attenuate the development of PD in T2DM patients.

### 3.3 Crosstalk between oxidative stress and inflammation in PD and T2DM

The inflammation process in the human body plays a central role in the pathogenesis of many chronic diseases including T2DM and PD.^121^ The oxidative burst of inflammatory cells during inflammation increases the production and accumulation of ROS in addition to mitochondrial dysfunction.^122^, ^123^ ROS regulates various types of kinases and transcription factors like NF‐κB which is related to the activation of pro‐inflammatory genes.^124^ The exact crosstalk between pro‐inflammatory markers and ROS in terms of pathogenesis and development of chronic diseases is still not fully elucidated. ROS, at high concentration, leads to damage in DNA, proteins and lipids causing injury and consequent inflammation on the cellular and tissue level which can lead to inflammation, premature aging disorders and several disease states.^125^ The relationship between ROS and pro‐inflammatory markers is directly interactive.^125^ A considerable body of experimental evidence has shown that ROS activates NF‐κB, which results in the transcriptional activation of inflammatory genes.^126^ There is a strong correlation between PD, redox imbalance and low‐grade chronic inflammation.^127^ Elevated levels of several inflammatory mediators and oxidised biomolecules are found in the CSF of PD patients, as well as in post‐mortem samples of SN from PD patients.^128^ Dopaminergic neurons are subjected to high levels of ROS generated from different sources including dopamine degradation, mitochondrial electron transport chain and oxidative modification of α‐synuclein.^129^ In the PD brain, the sustained activity of microglial NADPH oxidase exerts pathological effects both by direct ROS damage and by triggering inflammatory cytokine signalling that results in a vicious circle of neuronal damage.^130^ As well, aggregated α‐synuclein stimulates NADPH oxidase, eventually contributing to dopaminergic damage.^131^ Besides, astrocytes and microglia are activated in response to α‐synuclein‐induced inflammation leading to the generation of ROS and pro‐inflammatory cytokines in a vicious cycle.^132^ Higher oxidative stress and inflammation in T2DM promotes the peripheral and local activation of the NF‐κB and the NLRP3 inflammasome leading to neuroinflammation which involved in PD neuropathology.^133^

## 4 TARGETING OF NF‐κB/NLRP3 INFLAMMASOME AXIS IN T2DM AND PD

Targeting peripheral inflammation and neuroinflammation is integral in the attenuation of neurological complications including the development and progression of PD in T2DM. A cohort study on 64,166 T2DM and non‐T2DM patients on sulfonylureas alone or in combination with metformin followed for the development of PD showed that PD was increased by 2.2‐fold in T2DM, and sulfonylureas increased this risk by 57% which eliminated by combination with metformin.^134^ This finding suggests a protective role of metformin against the development of PD in T2DM patients. Metformin, which is currently used as a first‐line therapy for T2DM has recently been demonstrated to exert a neuroprotective role in several neurodegenerative disorders including PD.^135^ Metformin has been shown to inhibit α‐synuclein phosphorylation and aggregation, prevent mitochondrial dysfunction, attenuate oxidative stress and modulate autophagy mainly *through* AMP‐activated protein kinase (AMPK) activation, as well as prevent neurodegeneration and neuroinflammation.^135^ Metformin has been shown to ameliorate motor and cognitive dysfunction, by inhibiting α‐synuclein aggregation.^135^ Together, the neuroprotective effects of metformin in PD pathogenesis present a novel promising therapeutic strategy that might overcome the limitations of current PD treatment. NF‐kB is increased in a variety of tissues with aging, thus the inhibition of NF‐kB leads to delayed onset of aging‐related symptoms and pathologies such as diabetes, atherosclerosis, and PD.^136^ In virtue of its antioxidant, and anti‐inflammatory properties, metformin has become a possible candidate drug, improving in the context of aging and aging‐related diseases by inhibiting the expression of the NF‐kB gene, and eliminating the susceptibility to common diseases.^136^ Likewise, metformin has been reported to inhibit NLRP3 by activating autophagy through the AMPK‐dependent pathway.^137^ Particularly, AMPK activation attenuates NLRP3 inflammasome upregulation in some pathological processes, such as diabetes, pain, ischemic stroke, and endoplasmic reticulum stress. In addition, previous studies indicated that autophagy could downregulate the NLRP3 inflammasome via the mTOR signalling pathway.^138^ Moreover, metformin can exert a neuroprotective effect by neuroinflammation by attenuating the expression of the inflammatory signaling pathway.^139^ These findings suggest that metformin via the inhibition of the NF‐kB/ NLRP3 inflammasome axis may attenuate the development and progression of PD in T2DM patients.

Furthermore, DPP4 inhibitors like sitagliptin are commonly used in the management of T2DM and can reduce the propagation of neuroinflammation in different neurodegenerative disorders like AD and PD.^140^ DPP4 inhibitors repress Aβ accumulation, tau hyper‐phosphorylation, neuroinflammation, mitochondrial dysfunction and ROS formation, resulting in the inhibition of cognitive impairment.^140^ At a molecular level, insulin signaling impairment, abnormally higher activity of glycogen synthase kinase‐3 (GSK‐3) and the subsequent deregulated protein phosphorylation have been detected both in AD and T2DM patients.^141^ Consequently, it has been proposed that pharmacological agents against T2DM could also be beneficial for the prevention and/or treatment of AD. In this bargain, the association between T2DM and PD extends to antidiabetic drugs. Small clinical trials repurposing antidiabetic drugs in PD have yielded positive results with exenatide a glucagon‐like‐peptide 1 receptor agonist, but not with pioglitazone.^142^ A recent prospective study found strong evidence for a lower incidence of PD in users of DPP4 inhibitors and GLP‐1 agonists compared to users of control medications, and an inverse association between the use of these drugs and the onset of PD.^143^ Furthermore, these results were seen in both short‐term and long‐term >3 years users of GLP‐1 agonists and DPP4 inhibitors. While glitazone usage was associated with a lower PD risk, this finding was not significant in the adjusted models.^143^ Additional analyses showed that diabetes is associated with increased PD risk, the risk was highest for untreated diabetic patients and did not vary significantly between index and control medication groups for insulin‐user diabetics.^143^ It has been shown that DPP4 inhibitors can reduce vascular inflammation in metabolic disorders through the inhibition of NF‐kB.^144^, ^145^ Similarly, DPP4 inhibitors repress NLRP3 inflammasome activation in diabetic nephropathy and acute kidney injury.^144^, ^146^, ^147^

These observations proposed that antidiabetic agents can mitigate inflammatory disorders which are implicated in the pathogenesis of PD.

## 5 CONCLUSION

PD is one of the most common neurodegenerative disease due to the degeneration of dopaminergic neurons in the SN. Genetic predisposition and immune dysfunction are involved in the pathogenesis of PD. Besides, T2DM is linked with inflammatory disorders due to hyperglycaemia‐induced oxidative stress and the release of pro‐inflammatory cytokines. Therefore, T2DM may increase PD risk through the activation of inflammatory signaling pathways including NF‐κB and the NLRP3 inflammasome which impair insulin signaling and increase the risk for the development of brain IR a hallmark of PD neuropathology. Taken together, NF‐κB/NLRP3 inflammasome axis could be the causal pathway in the development of PD in T2DM patients. Targeting of this axis by anti‐inflammatory agents may prevent the development of PD in T2DM patients.

## 6 FUTURE THERAPEUTIC PERSPECTIVES

Targeting the inflammatory signaling pathways including NF‐κB and the NLRP3 inflammasome in T2DM patients may reduce future PD risk. A recent study observed that insulin‐sensitising drug metformin improves glucose homeostasis by inhibiting the central and peripheral NF‐κB and the NLRP3 inflammasome signaling pathways.^148^ Therefore, through inhibition of the central NF‐κB and the NLRP3 inflammasome signaling pathways, metformin could be effective in the management of PD.^135^ As well, metformin has a neuroprotective role against PD neuropathology through different mechanisms including autophagy upregulation, degradation of pathological α‐synuclein and regulation of mitochondrial functions.^149^ Furthermore, DPP4 inhibitors which are used in the management of T2DM can attenuate neuroinflammation and degeneration of dopaminergic neurons in the SN by inhibiting NF‐κB and the NLRP3 inflammasome signaling pathways.^146^, ^150^ Therefore, inhibition of NF‐κB and the NLRP3 inflammasome signaling pathways by antidiabetic agents' metformin and DPP4 inhibitors could be a potential therapeutic perspectives to prevent the development of PD in T2DM patients.

## AUTHOR CONTRIBUTIONS

**Mohammed Alrouji:** Validation (equal); visualization (equal). **Hayder M. Al‐kuraishy:** Conceptualization (equal); writing – original draft (equal). **Ali I. Al‐Gareeb:** Conceptualization (equal); writing – original draft (equal). **Athanasios Alexiou:** Writing – review and editing (equal). **Marios Papadakis:** Supervision (equal); writing – review and editing (equal). **Majid S. Jabir:** Writing – review and editing (equal). **Hebatallah M. Saad:** Project administration (equal); writing – review and editing (equal). **Gaber El‐Saber Batiha:** Writing – review and editing (equal).

## 7 FUNDING INFORMATION

This work was supported by the University of Witten‐Herdecke Germany.

## CONFLICT OF INTEREST STATEMENT

The authors declare no conflict of interest.

## Data Availability

Data sharing is not applicable to this article as no new data were created or analysed in this study.
